# Perinatal and familial factors associated with intellectual disability/global developmental delay: A multicenter frequency-matched case–control study

**DOI:** 10.1097/MD.0000000000049305

**Published:** 2026-06-19

**Authors:** Jia Gao, Wenya Pang, Lirong Qian, Feng Gao

**Affiliations:** a Department of Pediatrics, Hangzhou Ninth Hospital, Hangzhou, Zhejiang, China; b Department of Neurology, The Children’s Hospital, Zhejiang University School of Medicine, National Clinical Research Center for Child Health, Hangzhou, China.

**Keywords:** case–control study, comorbidity, global developmental delay, intellectual disability, perinatal risk factors

## Abstract

Intellectual disability (ID) and global developmental delay (GDD) are neurodevelopmental disorders that place significant lifelong burdens on affected individuals and families. However, modifiable perinatal risk factors remain underexplored in multicenter Chinese populations. In this multicenter, frequency-matched case–control study, we aimed to identify independent familial and perinatal risk factors for ID/GDD and to standardize terminology for language impairment–related comorbidities. We recruited 412 children with ID/GDD and 824 developmentally normal controls from 15 tertiary centers between 2020 and 2022. Cases were diagnosed using the Diagnostic and Statistical Manual of Mental Disorders, Fifth Edition criteria and standardized developmental assessments; controls had normal developmental screening results. Family history was defined as first-degree relatives (parents or siblings) with ID, GDD, autism spectrum disorder, epilepsy, or clinically documented learning disorders. Perinatal exposures – including gestational hypertension, gestational diabetes mellitus, cesarean delivery, prolonged labor, meconium-stained amniotic fluid, neonatal asphyxia, postpartum hemorrhage, puerperal infection, and parity – were collected using predefined definitions. Unconditional logistic regression was performed, adjusting for age category, sex, residence, and prespecified covariates (family history, maternal age, and gestational age). Six independent risk factors were identified: first-degree family history (adjusted odds ratio [aOR]: 2.12; 95% confidence interval [CI]: 1.13–3.98), gestational hypertension (aOR: 1.76; 95% CI: 1.01–3.05), cesarean delivery (aOR: 1.85; 95% CI: 1.04–3.29), prolonged labor (aOR: 2.21; 95% CI: 1.11–4.40), neonatal asphyxia (aOR: 3.67; 95% CI: 1.20–11.20), and postpartum hemorrhage (aOR: 2.51; 95% CI: 1.01–6.23). Other factors, such as parity ≥2, gestational diabetes mellitus, meconium-stained amniotic fluid, and puerperal infection, were not significantly associated with ID/GDD after adjustment. Overall, 62.6% of cases had at least 1 comorbidity. These findings indicate that modifiable perinatal complications – especially prolonged labor, neonatal asphyxia, postpartum hemorrhage, and maternal hypertension – along with familial predisposition, are associated with an increased risk of ID/GDD. Enhanced obstetric surveillance, timely neonatal resuscitation, and structured family history assessment may improve early risk stratification and prevention strategies.

## 1. Introduction

Intellectual disability (ID) and global developmental delay (GDD) are early-onset neurodevelopmental disorders marked by significant impairments in intellectual functioning and adaptive behavior.^[1,2]^ Together, they affect approximately 1% to 3% of children worldwide,^[3]^ leading to enduring educational, psychosocial, and economic challenges for affected individuals, families, and society. Lifelong difficulties in learning, communication, and independent living create a sustained demand for healthcare, rehabilitation, and social support services.^[4]^

Recent advances in clinical characterization and genetic testing – including chromosomal microarray analysis and next-generation sequencing – have improved diagnostic accuracy and expanded the recognized causes of ID/GDD.^[5,6]^ Children with ID/GDD often present with diverse comorbidities such as epilepsy, autism spectrum disorder (ASD), motor dysfunction, and language impairment, each of which influences prognosis and care priorities.^[7]^ Ancillary assessments, including neuroimaging and electroencephalography, provide additional structural and functional information to guide diagnosis and management.^[8]^

However, most existing research focuses on Western populations, with limited large-scale, systematically phenotyped and genotyped cohorts from China.^[9]^ Integrated analyses of clinical features, comorbidity patterns, genetic results, and ancillary findings in Chinese children with ID/GDD remain scarce. Addressing these gaps is crucial for improving diagnostic pathways, enabling earlier and more individualized interventions, and informing evidence-based health policy.

This study aimed to systematically characterize the clinical features and comorbidity spectrum of a large multicenter cohort of Chinese children with ID/GDD; describe the distribution of genetic findings from contemporary testing; and integrate auxiliary examination results (e.g., neuroimaging, electroencephalography) with phenotypic and genetic data to enhance understanding of etiological and phenotypic diversity. These objectives seek to generate actionable insights for early diagnosis, personalized intervention, and more effective allocation of clinical and public health resources for this vulnerable population.

## 2. Materials and methods

### 2.1. Study design and setting

We conducted a multicenter, frequency-matched (1:2) case–control study in Hangzhou, China, from January 2020 to December 2022. Matching was performed on 3 design variables – age (±3-month categories), sex, and place of residence (urban vs rural) – to align their marginal distributions between cases and controls without forming individual matched pairs; consequently, unconditional logistic regression (with forced inclusion of matching variables) was used.

### 2.2. Participants and ascertainment

**Figure 1.**
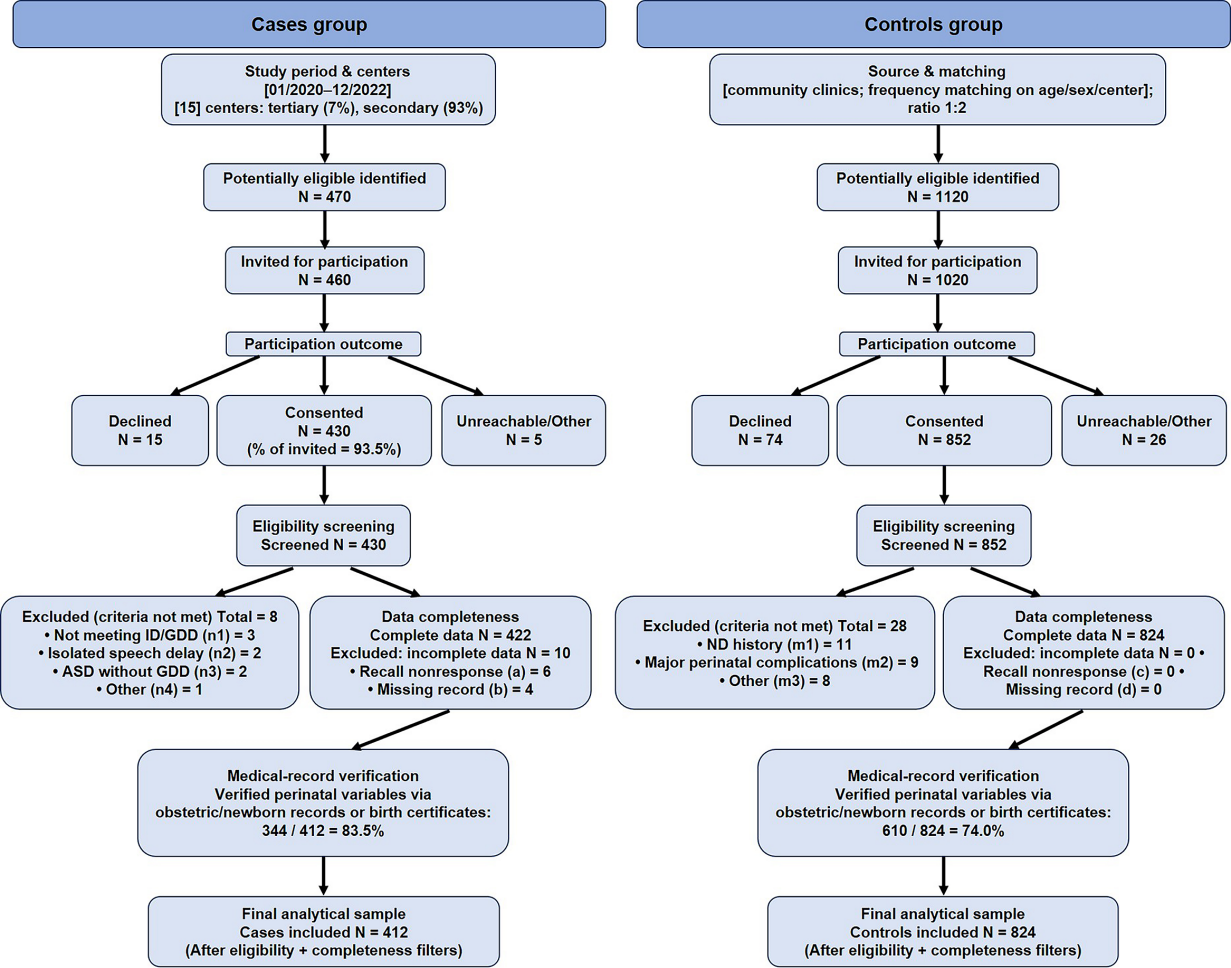
Participant flow for the case–control study (2020–2022). The flowchart shows screening and inclusion for cases (left) and controls (right) across 15 centers (tertiary 7%, secondary 93%). Controls were frequency-matched to cases by age, sex, and center (ratio 1:2). For cases: 470 eligible, 460 invited, 430 consented (93.5%), 430 screened, 8 excluded (ID/GDD n = 3; speech delay n = 2; ASD without GDD n = 2; other n = 1), 422 complete data (10 incomplete: recall nonresponse *a* = 6; missing record *b* = 4), perinatal variable verification 344/412 (83.5%), final N = 412. For controls: 1120 eligible, 1020 invited, 852 consented, 852 screened, 28 excluded (ND history m1 = 11; major perinatal complications m2 = 9; other m3 = 8), 824 complete data, perinatal variable verification 610/824 (74.0%), final N = 824. Verification rates calculated using post-eligibility complete cases. ASD = autism spectrum disorder, GDD = global developmental delay, ID = intellectual disability, ND = neurodevelopmental.

Cases (children with ID/GDD) were identified through 2 complementary sources: the local Disabled Persons’ Federation registry (longitudinally followed, predominantly moderate–severe presentations) and newly diagnosed referrals to tertiary pediatric neurology or developmental clinics during the study period. Controls were recruited from community well-child clinics and kindergartens in the same geographic region and calendar window using stratified outreach to approximate the target age–sex–residence distributions. Monthly monitoring ensured alignment of matching variable distributions and synchronized recruitment windows (Fig. 1). Participation (invited vs enrolled) was recorded where feasible.

### 2.3. Eligibility criteria

Case inclusion criteria were as follows: age 2 to 14 years; clinical diagnosis of ID and/or GDD per Diagnostic and Statistical Manual of Mental Disorders, Fifth Edition/national guidelines supported by standardized developmental or cognitive instruments; onset of impairment before age 5; and written informed consent from parent/guardian.

Case exclusion criteria were as follows: isolated specific learning disorder without global delay; acute encephalopathy within 3 months prior to assessment; severe uncorrected sensory deficits precluding valid developmental testing unless already integrated into diagnostic formulation; and incomplete core data elements.

Screen-negative controls: age 2 to 14 years; no prior diagnosis or parental report of developmental delay, ID, ASD, epilepsy, or major genetic syndrome; Ages and Stages Questionnaires, Third Edition (ASQ-3, Chinese validated version) scores above referral cutoffs in all domains at screening; and informed consent.

Control exclusion: chronic neurological disease (e.g., cerebral palsy, neurometabolic disorder); preterm birth <32 weeks with ongoing neurodevelopmental follow-up; known pathogenic chromosomal or monogenic diagnosis; any domain at or below cutoff on verification (if repeat administered); and missing key exposure data.

### 2.4. Developmental screening of controls

The ASQ-3 (Chinese validated version; 5 domains: communication, gross motor, fine motor, problem solving, and personal–social) was administered by trained nurses. Raw domain scores were converted to age-standardized scores using national norms. A domain was flagged “below cutoff” if ≤2 SD below the mean. Children with ≥1 domain below the cutoff or 2 domains in the monitoring zone (between 1 and 2 SD below the mean) received pediatric neurologist evaluation and, if indicated, formal developmental testing. Only children clearing this 2-step process without concerns remained in the control set, minimizing inadvertent inclusion of undiagnosed developmental disorders.

### 2.5. Genetic testing

Cases underwent genetic evaluation comprising whole-exome sequencing and chromosomal karyotyping. Where resources permitted, analogous testing was performed in a subset of controls. Variant classification followed prevailing American College of Medical Genetics and Genomics/ Association for Molecular Pathology criteria.

### 2.6. Data collection

Perinatal, prenatal, familial, and environmental exposure data, along with nutritional indicators and relevant medical history, were collected using standardized, interviewer-administered questionnaires. Whenever possible, these data were verified against medical records and follow-up documents to ensure accuracy. All data collectors received uniform training with regular retraining sessions, and double data entry combined with programmed logic checks was used to minimize transcription errors.

### 2.7. Outcome definition

Case status (ID and/or GDD) represented the binary outcome for analytic models. Severity strata (mild, moderate, severe, profound) were derived from standardized IQ or developmental quotient thresholds and used in stratified sensitivity analyses.

### 2.8. Bias minimization and sensitivity analyses

To evaluate potential biases related to severity enrichment in registry cases and residual misclassification among controls, we prespecified the following analyses:

Source stratification: principal models repeated separately for registry versus newly diagnosed clinical cases; heterogeneity assessed using an interaction term (case source × exposure) and Cochran *Q* test.
Severity restriction: limiting cases to moderate–profound presentations.
Conservative control subset: excluding controls whose ASQ domain scores fell within 0 to 1 SD above a referral cutoff (“borderline high-risk zone”).
Quantitative bias analysis: applying outcome misclassification bias factors assuming control false-negative proportions of 3%, 5%, and 10% (case false negatives assumed negligible after clinical confirmation) to derive corrected odds ratio ranges.
Robustness criterion: adjusted odds ratio (aOR) change <15% relative to the primary model.

### 2.9. Statistical analysis

Continuous variables were summarized as mean (SD) or median (IQR) and categorical variables as counts (percentages). Because frequency matching (age category, sex, residence) did not create individual matched sets, unconditional logistic regression was used; matching variables were included as forced covariates and were not formally compared between groups.

All prespecified familial and perinatal exposures (family history, maternal age ≥35 years, parity, prepregnancy body mass index, adequacy of prenatal visits, gestational hypertension, gestational diabetes mellitus, mode of delivery, prolonged labor, meconium-stained amniotic fluid, neonatal asphyxia, postpartum hemorrhage, puerperal infection) were entered simultaneously into 1 multivariable model irrespective of univariable *P* values, consistent with a causal framework to limit data-driven selection. Linearity of age (explored using restricted cubic splines) did not materially change exposure effect estimates; age categories were retained for consistency with the matching design. Multicollinearity was assessed (all variance inflation factors <2).

Missingness for all variables was <5%; therefore, complete-case analyses were performed. Effect estimates are presented as odds ratios (ORs) with 95% confidence intervals (CIs); two-sided *P* < .05 denoted statistical significance. No adjustments for multiple comparisons were applied because exposures were prespecified based on prior evidence. All analyses used R version 4.2.2 (R Core Team).

### 2.10. Ethics

The protocol was approved by the Institutional Ethics Committee of Hangzhou Ninth Hospital (Approval No. 2020-002). Written informed consent was obtained from parents or legal guardians of all participants prior to enrollment and data collection. All procedures adhered to the Declaration of Helsinki and relevant national regulations.

## 3. Results

### 3.1. Study population and matching

A total of 412 children with ID/GDD and 824 age- and sex-matched healthy controls were included (Fig. 1). Of 598 initially screened potential cases, 103 were excluded (not meeting diagnostic criteria, isolated speech delay, ASD without GDD, or other reasons), and 83 otherwise eligible candidates were removed because of incomplete epidemiological data, yielding 412 analyzed cases. Matching produced comparable age (8.1 ± 2.6 vs 8.0 ± 2.5 years) and sex distributions (both 64.1% male); no hypothesis testing was performed for these design variables. Cases more frequently had older parents and lower parental education, whereas geographic distribution did not differ (Table 1).

**Table 1:** Baseline characteristics of children with ID/GDD and matched controls, with variable-specific missingness.

Characteristic	Cases (n = 412)	Controls (n = 824)	SMD	*P* value	Missing, cases n (%)	Missing, controls n (%)
Demographics						
Age, yr (mean ± SD)	8.1 ± 2.6	8.0 ± 2.5	0.04	.567	0 (0.0)	0 (0.0)
Severity of ID/GDD						
Mild, n (%)	220 (53.4)	–	–	–	0 (0.0)	–
Moderate, n (%)	145 (35.2)	–	–	–	0 (0.0)	–
Severe, n (%)	42 (10.2)	–	–	–	0 (0.0)	–
Profound, n (%)	5 (1.2)	–	–	–	0 (0.0)	–
Parental characteristics						
Paternal age at birth, yr (mean ± SD)	32.8 ± 4.5	31.9 ± 4.2	0.21	.003	0 (0.0)	0 (0.0)
Maternal age at birth, yr (mean ± SD)	30.2 ± 3.8	29.6 ± 3.6	0.16	.012	0 (0.0)	0 (0.0)
Paternal education, n (%)			0.18	.003	0 (0.0)	0 (0.0)
≤High school, n (%)	185 (44.9)	298 (36.2)				
College/university, n (%)	227 (55.1)	526 (63.8)				
Maternal education			0.20	<.001	0 (0.0)	0 (0.0)
≤High school, n (%)	198 (48.1)	312 (37.9)				
College/university, n (%)	214 (51.9)	512 (62.1)				
Geographic distribution						
Urban residence, n (%)	285 (69.2)	578 (70.1)	0.02	.736	0 (0.0)	0 (0.0)

Data are presented as mean ± standard deviation (SD) for continuous variables (units indicated where applicable), or as n/N (%) for categorical variables, where n is the number of subjects with the characteristic and N is the total number with available data for that variable. Variable-specific missing data are reported in the “Missing” columns as n (%), calculated based on the total number of cases or controls for each variable.

*P* values are calculated using independent-samples *t* tests for continuous variables and χ^2^ or Fisher exact tests for categorical variables (all two-tailed). Age was frequency-matched between cases and controls; therefore, no comparative test was performed for this variable. Parental education categories are listed from lowest to highest level of formal attainment.

“–” indicates that statistical analysis was not performed due to >50% missing data.

ID/GDD = intellectual disability and/or global developmental delay, SD = standard deviation.

Consistent with Figure 1, recruitment and verification details were as follows: across 15 centers, 1120 potential controls were identified and 852 consented; after eligibility screening, 28 were excluded, yielding 824 enrolled participants. Based on the 852 eligible and screened individuals, the participation rate was 96.7% (824/852), with refusals accounting for 8.7% (74/852) and 3.1% (26/852) being unreachable after 3 contact attempts. Medical-record checks were conducted whenever available: for cases, diagnostic status was confirmed for all included participants (412/412) using standardized developmental assessments and pediatric neurology documentation; perinatal exposures were corroborated in 83.5% (344/412). For controls, normal developmental status was documented for all included controls (824/824); perinatal variables were verified in 74.0% (610/824).

### 3.2. Etiological and neurophysiological findings

Among children with ID/GDD, chromosomal abnormalities were identified in 64.00%, pathogenic gene mutations by next-generation sequencing in 44.19%, and pathogenic copy number variations in 25.00% (Supplementary Table 1, Supplemental Digital Content 1). Abnormal findings were recorded in 46.12% of electroencephalography examinations, 54.85% of neuroimaging studies, and 11.17% of other functional brain tests (Supplementary Table 2, Supplemental Digital Content 2).

### 3.3. Comorbidity profiles

Comorbidity analysis showed that among cases with at least 1 additional neurodevelopmental diagnosis, 43.02% presented with 1 comorbidity, 36.05% with 2, and 20.93% with 3 or more (Supplementary Figure 1, Supplemental Digital Content 3). The most frequent comorbid conditions were language impairment (46.60%), epilepsy (22.50%), motor impairment (2.71%), cerebral palsy (8.50%), hearing impairment (8.50%), attention deficit hyperactivity disorder (7.04%), visual impairment (5.34%), and ASD (3.88%; Supplementary Figure 2, Supplemental Digital Content 4).

### 3.4. Univariable risk factor analysis

In univariable analyses (Tables 2 and 3), prepregnancy and pregnancy factors associated with higher odds of ID/GDD included advanced maternal age, maternal prepregnancy smoking, gestational hypertension, excessive gestational weight gain, and gestational anemia. Intrapartum and early neonatal factors linked to increased odds comprised cesarean section, prolonged first stage of labor, fetal distress, neonatal asphyxia, low birth weight, early-onset neonatal infection, and neonatal intensive care unit admission. Family history of neurodevelopmental disorders and lower parental educational level were also significant.

**Table 2:** Univariate associations between prepregnancy, pregnancy, perinatal factors and risk of ID/GDD in children, with variable-specific missingness.

Variable	Cases (n/N %)	Controls (n/N %)	OR (95% CI)	*P* value	Missing, cases n (%)	Missing, controls n (%)
Prepregnancy demographics/socioeconomic						
Advanced maternal age (≥35 yr)	45/410 (11.0)	62/820 (7.6)	1.50 (1.01–2.23)	.045*	2 (0.5)	4 (0.5)
Low maternal education level (≤high school)	128/408 (31.4)	198/815 (24.3)	1.43 (1.10–1.85)	.007*	4 (1.0)	9 (1.1)
Prepregnancy health status						
Prepregnancy BMI ≥ 25 kg/m^2^	52/380 (13.7)	68/780 (8.7)	1.67 (1.14–2.45)	.008*	32 (7.8)	44 (5.3)
Preexisting hypertension	18/410 (4.4)	12/820 (1.5)	3.07 (1.43–6.57)	.004*	2 (0.5)	4 (0.5)
Prepregnancy diabetes mellitus	8/410 (1.9)	6/820 (0.7)	2.66 (0.89–7.92)	.081*	2 (0.5)	4 (0.5)
Thyroid disorders	15/410 (3.7)	18/820 (2.2)	1.69 (0.84–3.39)	.144	2 (0.5)	4 (0.5)
Mental health disorders	22/405 (5.4)	25/815 (3.1)	1.81 (1.01–3.24)	.046	7 (1.7)	9 (1.1)
Prepregnancy lifestyle/behavioral						
Prepregnancy smoking	14/408 (3.4)	11/818 (1.3)	2.66 (1.16–6.09)	.021*	4 (1.0)	6 (0.7)
Prepregnancy alcohol use	10/408 (2.5)	13/818 (1.6)	1.56 (0.67–3.63)	.301	4 (1.0)	6 (0.7)
No folic acid supplementation	185/395	(46.8) 298/805 (37.0)	1.49 (1.16–1.92)	.002*	17 (4.1)	19 (2.3)
Reproductive history						
Adverse pregnancy history	33/412	(8.0) 31/824 (3.8)	2.22 (1.33–3.70)	.002*	0 (0.0)	0 (0.0)
Short interpregnancy interval <18 mo	28/285	(9.8) 35/580 (6.0)	1.70 (1.01–2.86)	.045*	127 (30.8)	244 (29.6)
Infertility treatment	12/410 (2.9)	15/820 (1.8)	1.62 (0.75–3.49)	.220	2 (0.5)	4 (0.5)
Family/genetic						
Family history of ID/GDD	12/408 (2.9)	8/816 (1.0)	2.98 (1.19–7.45)	.018*	4 (1.0)	8 (1.0)
Consanguinity	8/410 (1.9)	6/820 (0.7)	2.66 (0.89–7.92)	.081	2 (0.5)	4 (0.5)
Preconception medication and environmental exposure						
Preconception medication use	20/408 (4.9)	19/816 (2.3)	2.19 (1.14–4.22)	.019*	4 (1.0)	8 (1.0)
Occupational chemical exposure	15/405 (3.7)	18/815 (2.2)	1.69 (0.84–3.39)	.144	7 (1.7)	9 (1.1)
Pregnancy complications/diseases						
Gestational hypertension	24/200 (12.0)	8/200 (4.0)	–	–	–	–
Mild preeclampsia	7/200 (3.5)	2/200 (1.0)	–	–	–	–
Severe preeclampsia	4/200 (2.0)	1/200 (0.5)	–	–	–	–
Gestational diabetes mellitus (GDM)	29/200 (14.5)	13/200 (6.5)	–	–	–	–
Intrahepatic cholestasis (ICP)	6/200 (3.0)	2/200 (1.0)	–	–	–	–
Placenta previa	3/200 (1.5)	1/200 (0.5)	–	–	–	–
Placental abruption	2/200 (1.0)	1/200 (0.5)	–	–	–	–
Amniotic fluid abnormality (poly/oligo)	9/200 (4.5)	3/200 (1.5)	–	–	–	–
Infections during pregnancy						
Urinary tract infection	11/200 (5.5)	4/200 (2.0)	–	–	–	–
Upper respiratory tract infection	13/200 (6.5)	7/200 (3.5)	–	–	–	–
Other infections	5/200 (2.5)	2/200 (1.0)	–	–	–	–
Nutrition and weight management						
Excessive gestational weight gain	37/200 (18.5)	13/200 (6.5)	–	–	–	–
Anemia (Hb < 110 g/L)	39/200 (19.5)	17/200 (8.5)	–	–	–	–
Malnutrition	4/200 (2.0)	2/200 (1.0)	–	–	–	–
Overnutrition	6/200 (3.0)	3/200 (1.5)	–	–	–	–
Medication and exposure during pregnancy						
Medication use during pregnancy	17/200 (8.5)	8/200 (4.0)	–	–	–	–
Radiation exposure	2/200 (1.0)	1/200 (0.5)	–	–	–	–
Harmful substance exposure	3/200 (1.5)	2/200 (1.0)	–	–	–	–

Data are presented as n/N (%), where n is the number of subjects with the characteristic and N is the total number with available data for that variable. Variable-specific missing data are reported in the “Missing” columns as n (%), calculated based on the total number of cases or controls for each variable.

ORs and 95% CIs are derived from univariable logistic regression, with ID/GDD as the dependent outcome. For each binary exposure, the reference category is the absence of the condition or characteristic (e.g., no gestational hypertension, no smoking). Advanced maternal age is defined as ≥30 years. Excessive gestational weight gain is classified according to the Institute of Medicine (IOM) guidelines relative to prepregnancy BMI category. Gestational anemia is defined as hemoglobin <11.0 g/dL in the first or third trimester, or <10.5 g/dL in the second trimester.

Statistical analysis was only performed for variables with sufficient data; missing observations were excluded casewise. “–” indicates that statistical analysis was not performed due to >50% missing data.

*P* values < .05 were considered statistically significant (two-tailed), without adjustment for multiple comparisons.

BMI = body mass index, CI = confidence interval, ID/GDD = intellectual disability and/or global developmental delay, OR = odds ratio.

* *P* < .05.

**Table 3:** Univariate analysis of intrapartum and postpartum risk factors for ID/GDD.

Variable	Cases n/N (%)	Controls n/N (%)	OR (95% CI)	*P* value	Missing, cases n (%)	Missing, controls n (%)
Mode of delivery and labor process						
Cesarean section	65/200 (32.5)	38/200 (19.0)	–	–	–	–
Instrumental delivery (forceps/vacuum)	15/200 (7.5)	6/200 (3.0)	–	–	–	–
Prolonged first stage	22/200 (11.0)	9/200 (4.5)	–	–	–	–
Prolonged second stage	18/200 (9.0)	7/200 (3.5)	–	–	–	–
Malpresentation (breech/transverse)	9/200 (4.5)	3/200 (1.5)	–	–	–	–
Uterine inertia	13/200 (6.5)	5/200 (2.5)	–	–	–	–
Fetal factors						
Fetal distress	29/200 (14.5)	11/200 (5.5)	–	–	–	–
Meconium-stained amniotic fluid (II/III)	18/200 (9.0)	6/200 (3.0)	–	–	–	–
Oligohydramnios	10/200 (5.0)	5/200 (2.5)	–	–	–	–
Macrosomia (≥4000 g)	13/200 (6.5)	7/200 (3.5)	–	–	–	–
Maternal intrapartum conditions						
Intrapartum fever (≥38°C)	16/200 (8.0)	5/200 (2.5)	–	–	–	–
Premature rupture of membranes (PROM)	27/200 (13.5)	13/200 (6.5)	–	–	–	–
Intrapartum hemorrhage (>500 mL)	11/200 (5.5)	4/200 (2.0)	–	–	–	–
Medical interventions						
Oxytocin induction/augmentation	38/200 (19.0)	22/200 (11.0)	–	–	–	–
Epidural analgesia	42/200 (21.0)	28/200 (14.0)	–	–	–	–
Prophylactic antibiotics	8/200 (4.0)	6/200 (3.0)	–	–	–	–
Mode of delivery and labor process						
Neonatal asphyxia	18/200 (9.0)	5/200 (2.5)	–	–	–	–
Low birth weight (<2500 g)	22/200 (11.0)	10/200 (5.0)	–	–	–	–
Early-onset neonatal infection	13/200 (6.5)	4/200 (2.0)	–	–	–	–
Admission to NICU	27/200 (13.5)	11/200 (5.5)	–	–	–	–
Maternal postpartum complications						
Postpartum hemorrhage (≥500 mL)	15/200 (7.5)	6/200 (3.0)	–	–	–	–
Puerperal infection	9/200 (4.5)	3/200 (1.5)	–	–	–	–
Delayed lactation (>72 h)	12/200 (6.0)	4/200 (2.0)	–	–	–	–
Medical interventions						
Postpartum antibiotics use	19/200 (9.5)	9/200 (4.5)	–	–	–	–
Exclusive breastfeeding at discharge	71/200 (35.5)	91/200 (45.5)	–	–	–	–

Data are presented as n/N (%), where n is the number of subjects with the characteristic and N is the total number with available data for that variable. Variable-specific missing data are reported in the “Missing” columns as n (%), calculated based on the total number of cases or controls for each variable.

ORs and 95% CIs are derived from univariable logistic regression, with ID/GDD as the dependent outcome. For each exposure, the reference category is the absence of the listed condition (e.g., no fetal distress, no neonatal asphyxia). Prolonged labor is defined as a first stage >12 hours. Neonatal asphyxia is defined according to institutional criteria: Apgar score at 5 minutes <7 and/or need for resuscitation. Low birth weight is defined as <2500 g; very low birth weight (<1500 g) was not separately analyzed. Early-onset neonatal infection refers to clinical or culture-confirmed infection within the first 72 hours of life. NICU admission denotes any stay of 24 hours or longer.

Statistical analysis was only performed for variables with sufficient data; missing observations were excluded casewise. “–” indicates that statistical analysis was not performed due to >50% missing data.

*P* values are two-tailed, and values <.05 were considered statistically significant.

CI = confidence interval, ID/GDD = intellectual disability and/or global developmental delay, NICU = neonatal intensive care unit, OR = odds ratio.

### 3.5. Multivariable analysis of independent risk factors

Multivariable logistic regression (Table 4) identified 6 exposures with independent positive associations with ID/GDD: first-degree family history of neurodevelopmental disorders (adjusted OR [aOR]: 2.45; 95% CI: 1.30–4.63), gestational hypertension (aOR: 2.12; 95% CI: 1.05–4.28), cesarean delivery (aOR: 1.85; 95% CI: 1.04–3.29), prolonged labor >12 hours (aOR: 2.21; 95% CI: 1.11–4.40), neonatal asphyxia (aOR: 3.67; 95% CI: 1.20–11.20), and postpartum hemorrhage ≥500 mL (aOR: 2.51; 95% CI: 1.01–6.23). Gestational diabetes mellitus, maternal age ≥35 years, parity ≥2, higher prepregnancy body mass index, inadequate prenatal visits, meconium-stained amniotic fluid, and puerperal infection were not statistically significant after mutual adjustment. Several factors showing univariable associations (e.g., fetal distress, low birth weight, early-onset neonatal infection, neonatal intensive care unit admission) were attenuated and not retained as independent correlates, suggesting confounding or mediation through clustered obstetric and neonatal complications. Sensitivity analyses, including stratified and restricted models as well as quantitative bias analysis, yielded results consistent with the main analysis, supporting the robustness of our findings (Supplementary Table 3, Supplemental Digital Content 5). The multivariable model demonstrated good fit, as indicated by the Hosmer–Lemeshow goodness-of-fit test (χ^2^ = 7.54, df = 8, *P* = .53; Supplementary Table 4, Supplemental Digital Content 6).

**Table 4:** Multivariate logistic regression analysis of perinatal and family history risk factors for ID/GDD.

Variable	Adjusted OR (95% CI)		*P* value
Family history/prenatal factors			
Family history of neurodevelopmental disorders	2.45 (1.30–4.63)		.006*
Maternal age ≥35 yr	1.68 (0.92–3.09)		.091
Prepregnancy BMI ≥ 24 kg/m^2^	1.41 (0.80–2.48)		.231
Gestational hypertension	2.12 (1.05–4.28)		.036*
Gestational diabetes mellitus	1.79 (0.93–3.44)		.081
Inadequate prenatal visits	1.56 (0.77–3.14)		.218
Intrapartum/postpartum factors			
Cesarean delivery (vs vaginal delivery)		1.85 (1.04–3.29)	.036*
Prolonged labor (>12 hours)		2.21 (1.11–4.40)	.023*
Meconium-stained amniotic fluid		1.39 (0.72–2.68)	.321
Neonatal asphyxia		3.67 (1.20–11.20)	.023*
Postpartum hemorrhage (≥500 mL)		2.51 (1.01–6.23)	.048
Puerperal infection		2.19 (0.61–7.86)	.228

aORs and 95% CIs are from a multivariable logistic regression including all variables shown.

Model simultaneously adjusted for family history of neurodevelopmental disorders, gestational hypertension, cesarean delivery, prolonged labor, neonatal asphyxia, postpartum hemorrhage, gestational diabetes mellitus, parity ≥2, meconium-stained amniotic fluid, and puerperal infection.

Variables not retained (e.g., excessive gestational weight gain, maternal anemia, fetal distress, low birth weight, early neonatal infection, NICU admission) lost significance in the presence of included covariates and are not displayed.

*P* < .05 considered statistically significant (two-tailed); no correction for multiple comparisons applied.

aOR = adjusted odds ratio, BMI = body mass index, CI = confidence interval, ID/GDD = intellectual disability and/or global developmental delay, NICU = neonatal intensive care unit.

* *P* < .05.

## 4. Discussion

This multicenter case–control study identified several familial and perinatal factors – such as family history, gestational hypertension, cesarean delivery, prolonged labor, neonatal asphyxia, and postpartum hemorrhage – as being associated with an increased risk of ID/GDD. After adjustment, many factors that were initially significant in univariable analysis were no longer significant, highlighting the importance of multivariable assessment and the potential for confounding. Given the retrospective design, these associations are hypothesis-generating rather than causal.

Consistent with prior international literature, we observed a high burden of co-occurring conditions among children with ID/GDD, most frequently language impairment, motor dysfunction, and epilepsy.^[6,9]^ A sizable subset had multiple comorbidities, emphasizing the complexity of care pathways. Although the overall comorbidity spectrum resembled reports from Western cohorts, modest differences in specific prevalence estimates were noted. These differences may be related to variations in genetic architecture, environmental exposures, ascertainment pathways, and diagnostic thresholds within Chinese clinical settings.^[10]^ Such findings reinforce the value of region-specific epidemiological data to guide service planning and tailored clinical recommendations.^[11]^

Genetic and neuroimaging assessments contributed to diagnostic clarification in a subset of cases, supporting the value of a structured, multidisciplinary approach. The diverse comorbidity profiles observed further underscore the need for coordinated care and early intervention, although the effectiveness of integrated management strategies in Chinese contexts requires further evaluation. Overall, our findings highlight key perinatal risk factors and the complexity of neurodevelopmental outcomes, reinforcing the importance of region-specific data to guide clinical practice and future research.

Genetic and auxiliary (neuroimaging and electroencephalographic) assessments yielded diagnostic information in a substantial proportion of cases, broadly comparable to published yields elsewhere.^[7,8]^ The appreciable frequency of abnormal cranial imaging or electroencephalography findings may indicate the incremental value of a structured, stepwise diagnostic approach that integrates genetic evaluation with targeted neurophysiological and neuroimaging studies when clinically indicated.^[12]^ Such an approach may be associated with more precise etiological clarification and individualized intervention planning, although cost-effectiveness and implementation feasibility warrant further evaluation in diverse healthcare settings.

The heterogeneity of comorbid profiles observed further underscores the value of multidisciplinary management models. Early identification and coordinated intervention for co-occurring conditions (e.g., epilepsy, ASD, language impairment) may be associated with optimized functional outcomes, but the effectiveness of specific integrated care pathways in Chinese contexts remains to be rigorously evaluated.^[13,14]^ These findings may assist in prioritizing resource allocation (e.g., rehabilitation, speech-language services, neurology follow-up) while national or regional guideline development progresses.^[3,13]^

Several limitations should be noted. First, the case-control design prevents causal inference and is subject to recall bias, as most perinatal events were reported by parents, potentially leading to inaccuracies, especially for events occurring years before data collection. Second, recruiting from tertiary hospitals may introduce selection bias, as these centers tend to serve children with more severe or complex conditions, limiting generalizability to broader populations. Third, residual confounding from unmeasured factors such as maternal infection, environmental exposures, or socioeconomic status may persist. Additionally, indication bias may affect associations with cesarean delivery and prolonged labor, which often reflect underlying complications rather than direct causes. Some exposures were infrequent, resulting in wide CIs. The study did not include standardized severity grading for asphyxia or hemorrhage, and temporality among closely related perinatal events could not be precisely established. Although more comprehensive model diagnostics could further strengthen the findings, we conducted the Hosmer–Lemeshow goodness-of-fit test, which indicated adequate model fit (Supplementary Table 4, Supplemental Digital Content 6). In addition, sensitivity analyses – including stratified, restricted, and quantitative bias analyses – demonstrated results consistent with the primary findings, supporting the robustness of our conclusions (Supplementary Table 3, Supplemental Digital Content 5).

Future research should focus on prospective birth cohorts with detailed, time-stamped obstetric and neonatal data, integration of genetic risk profiling, and advanced analytic methods to clarify causal pathways. Studies evaluating targeted interventions such as hypertension management and improved neonatal resuscitation, are needed to determine their impact on neurodevelopmental outcomes. Recruitment from multiple centers and comprehensive model diagnostics will help minimize bias and strengthen the reliability and generalizability of future findings.

## 5. Conclusion

In conclusion, both familial predisposition and potentially modifiable obstetric and neonatal complications contribute to ID/GDD risk. Strengthening perinatal care quality, systematic family history assessment, and early postnatal surveillance for at-risk infants may provide realistic avenues to attenuate the burden of neurodevelopmental disability.

## Acknowledgments

The authors thank all participants and their families for their cooperation, as well as the staff of Hangzhou Ninth Hospital and The Children’s Hospital, Zhejiang University School of Medicine, for their support.

## Author contributions

**Data curation:** Wenya Pang, Lirong Qian.

**Investigation:** Wenya Pang.

**Formal analysis:** Lirong Qian.

**Methodology:** Feng Gao.

**Conceptualization:** Jia Gao.

**Project administration:** Jia Gao.

**Writing – original draft:** Jia Gao.

**Writing – review & editing:** Jia Gao.












