# Supplementary material for: Perinatal and familial factors associated with intellectual disability/global developmental delay: A multicenter frequency-matched case–control study
Source: Medicine (Baltimore). 2026 Jun 19;105(25):e49305. doi: 10.1097/MD.0000000000049305 (PMC13286462; doi:10.1097/MD.0000000000049305)
Supplement: Supplementary file 1 [file medi-105-e49305-s001.docx]

Suppl-Table 1 Summary of genetic testing results in children with ID/GDD.

| ​Testing Item | Number  tested (N) | Abnormal number  (N) | Abnormal percentage (%) |
| --- | --- | --- | --- |
| Chromosomal Abnormalities (Karyotype Analysis) | 78 | 50 | 64.00% |
| Gene Mutations  (Next-Generation Sequencing) | 43 | 19 | 44.19% |
| Pathogenic Copy Number Variations (CNV) | 20 | 4 | 20.00% |

Data are presented as n/total (%). Percentages are calculated using the number of tested participants as denominator.

Chromosomal abnormalities include numerical and structural variants detected by standard karyotyping.

Pathogenic/likely pathogenic gene mutations identified via next‑generation sequencing (panel / exome).

Copy number variations (CNVs) detected by chromosomal microarray.

Participants may harbor more than one genetic finding; categories are not mutually exclusive unless stated.

Abbreviations: CNV, copy number variation; NGS, next‑generation sequencing.
