# Supplementary material for: Perinatal and familial factors associated with intellectual disability/global developmental delay: A multicenter frequency-matched case–control study
Source: Medicine (Baltimore). 2026 Jun 19;105(25):e49305. doi: 10.1097/MD.0000000000049305 (PMC13286462; doi:10.1097/MD.0000000000049305)
Supplement: Supplementary file 2 [file medi-105-e49305-s002.docx]

Suppl-Table 2. Summary of electrophysiological, neuroimaging, and functional brain test findings in children with ID/GDD.

| ​Testing Item | Number  tested (N) | Abnormal number  (N) | | Abnormal percentage (%) |
| --- | --- | --- | --- | --- |
| Electroencephalogram | 190 | 46 | 24.21% | |
| Electromyogram | 62 | 8 | 12.90% | |
| Brainstem auditory evoked potential | 46 | 14 | 30.43% | |
| Cranial imaging examination (CT/MR) | 226 | 154 | 68.14% | |

Abbreviations: CT, computed tomography; MR, magnetic resonance; ID/GDD, intellectual disability/global developmental delay.

Data are shown as n (%), unless otherwise indicated.
