# Supplementary figures and images for: Perinatal and familial factors associated with intellectual disability/global developmental delay: A multicenter frequency-matched case–control study
Source: Medicine (Baltimore). 2026 Jun 19;105(25):e49305. doi: 10.1097/MD.0000000000049305 (PMC13286462; doi:10.1097/MD.0000000000049305)

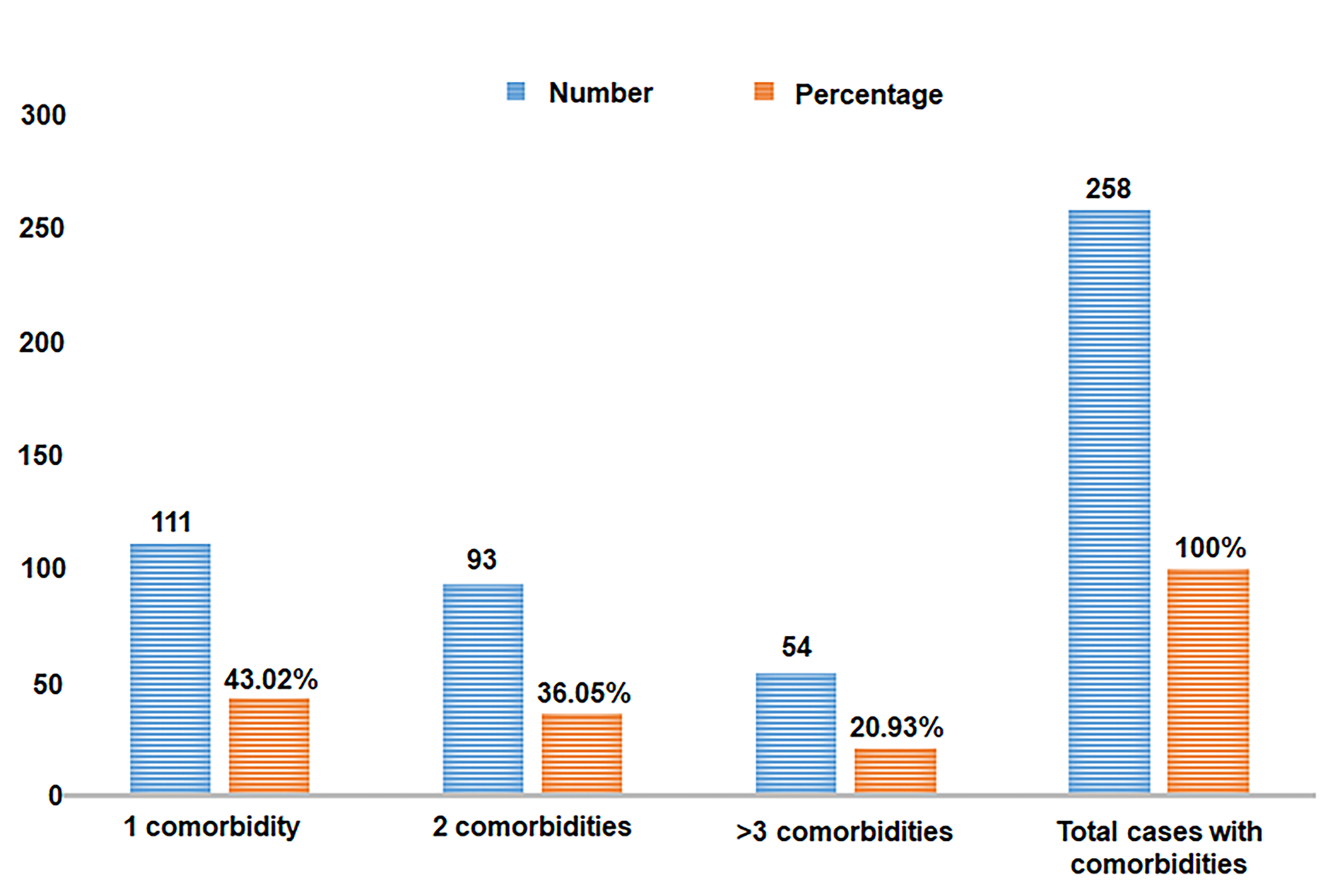

Supplement: Supplementary file 3 [file medi-105-e49305-s003.tif]

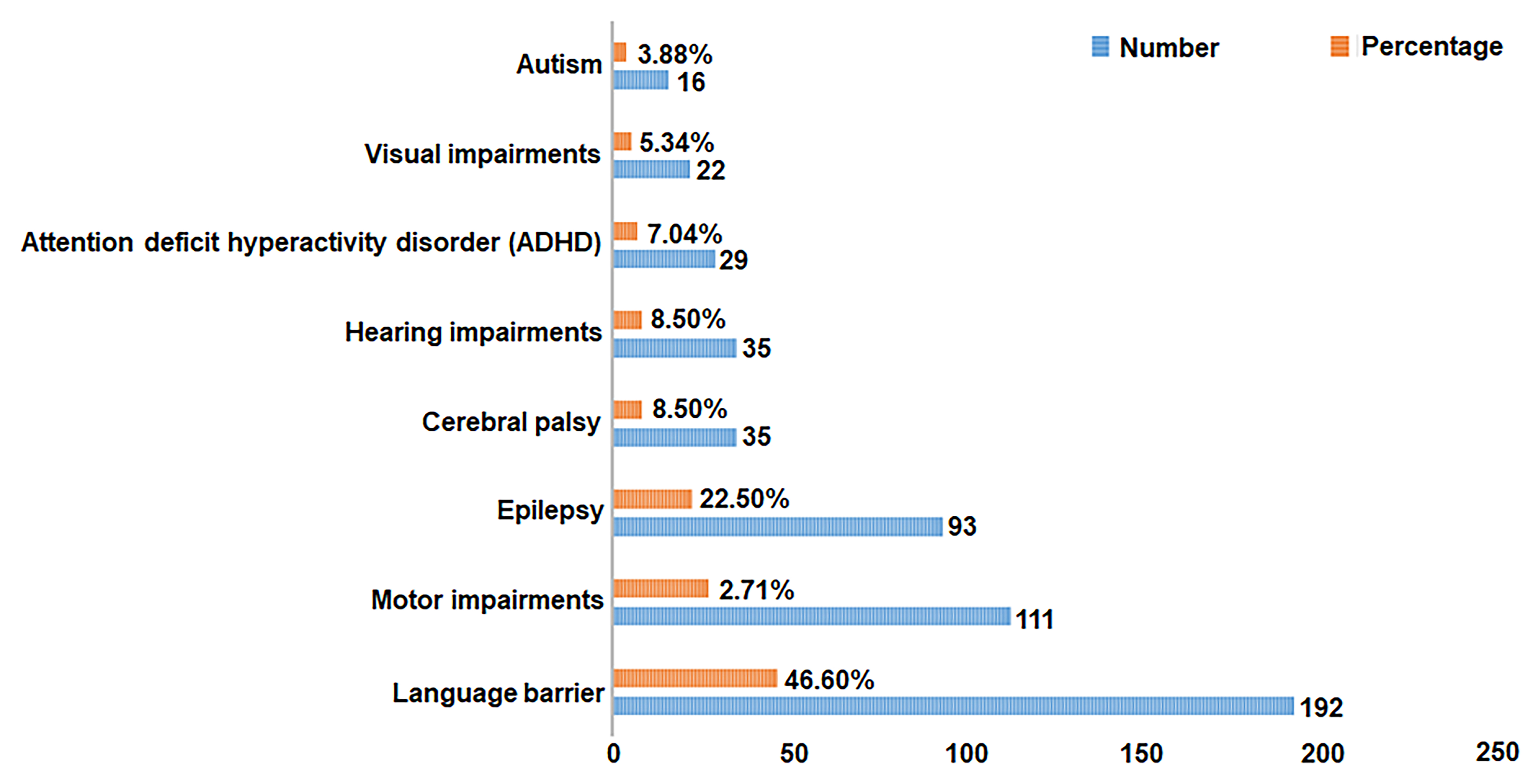

Supplement: Supplementary file 4 [file medi-105-e49305-s004.tif]
