# Supplementary material for: Perinatal and familial factors associated with intellectual disability/global developmental delay: A multicenter frequency-matched case–control study
Source: Medicine (Baltimore). 2026 Jun 19;105(25):e49305. doi: 10.1097/MD.0000000000049305 (PMC13286462; doi:10.1097/MD.0000000000049305)
Supplement: Supplementary file 5 [file medi-105-e49305-s005.docx]

Suppl Table 3. Sensitivity Analysis of Intrapartum and Postpartum Factors.

| Risk Factor | Main Analysis (adjusted OR, 95% CI, P) | Sensitivity Analysis (adjusted OR, 95% CI, P) |
| --- | --- | --- |
| Family history of neurodevelopmental disorders | 2.45 (1.30–4.63), 0.006 | 2.21 (1.12–4.38), 0.015 |
| Gestational hypertension | 2.12 (1.05–4.28), 0.036 | 1.97 (0.98–4.00), 0.042 |
| Cesarean delivery (vs. vaginal delivery) | 1.85 (1.04–3.29), 0.036 | 1.72 (0.98–3.15), 0.045 |
| Prolonged labor (>12 hours) | 2.21 (1.11–4.40), 0.023 | 2.00 (1.03–4.10), 0.020 |
| Neonatal asphyxia | 3.67 (1.20–11.20), 0.023 | 3.35 (1.09–10.00), 0.031 |
| Postpartum hemorrhage (≥500 ml) | 2.51 (1.01–6.23), 0.048 | 2.78 (0.98–6.10), 0.040 |

Neonatal asphyxia defined as Apgar score ≤7 at 1 or 5 minutes.

Postpartum hemorrhage defined as estimated blood loss ≥500 ml within 24 hours after delivery.

aOR: adjusted odds ratio; CI: confidence interval.Variables with P < 0.05 are considered statistically significant

Sensitivity analysis was performed by excluding cases with missing data to assess the robustness of the main results.
