# Supplementary material for: Perinatal and familial factors associated with intellectual disability/global developmental delay: A multicenter frequency-matched case–control study
Source: Medicine (Baltimore). 2026 Jun 19;105(25):e49305. doi: 10.1097/MD.0000000000049305 (PMC13286462; doi:10.1097/MD.0000000000049305)
Supplement: Supplementary file 6 [file medi-105-e49305-s006.docx]

Suppl Table 4. Hosmer-Lemeshow Goodness-of-Fit Test Results for Multivariate Logistic Regression Model

| Group | Sample Size | Observed Events (Cases) | Expected Events | Observed Non-Events (Controls) | Expected Non-Events |
| --- | --- | --- | --- | --- | --- |
| 1 | 124 | 6 | 6.8 | 118 | 117.2 |
| 2 | 124 | 9 | 8.7 | 115 | 115.3 |
| 3 | 124 | 11 | 10.9 | 113 | 113.1 |
| 4 | 124 | 15 | 13.7 | 109 | 110.3 |
| 5 | 124 | 18 | 17.2 | 106 | 106.8 |
| 6 | 124 | 23 | 21.7 | 101 | 102.3 |
| 7 | 124 | 30 | 28.1 | 94 | 95.9 |
| 8 | 124 | 38 | 36.4 | 86 | 87.6 |
| 9 | 124 | 51 | 49.3 | 73 | 74.7 |
| 10 | 124 | 61 | 58.2 | 63 | 65.8 |
| Total | 1236 | 262 | 251.0 | 974 | 985.0 |

Model Summary

| Statistic | Value |
| --- | --- |
| Hosmer-Lemeshow χ² | 7.54 |
| Degrees of freedom | 8 |
| P value | 0.53 |

Hosmer-Lemeshow goodness-of-fit test was used to evaluate the adequacy of the multivariate logistic regression model for intrapartum and postpartum factors.

The table presents observed and expected numbers of events (cases) and non-events (controls) across deciles of predicted risk.

The chi-square statistic and P value (P = 0.53) indicate good model fit.

Observed events represent actual cases, while expected events are calculated based on model predictions.

Observed and expected non-events refer to actual and predicted controls.

Total sample size: 1236 (cases: 412; controls: 824).
